# A recurrent homozygous missense *DPM3* variant leads to muscle and brain disease

**DOI:** 10.1111/cge.14208

**Published:** 2022-08-19

**Authors:** Sara Nagy, Tracy Lau, Shahryar Alavi, Ehsan Ghayoor Karimiani, Jalal Vallian, Bobby G. Ng, Samaneh Noroozi Asl, Javad Akhondian, Amir Bahreini, Omid Yaghini, Prech Uapinyoying, Carsten Bonnemann, Hudson H. Freeze, Vajira H. W. Dissanayake, Nirmala D. Sirisena, Miriam Schmidts, Henry Houlden, Andres Moreno‐De‐Luca, Reza Maroofian

**Affiliations:** ^1^ MRC Centre for Neuromuscular Diseases UCL Queen Square Institute of Neurology London UK; ^2^ Department of Neurology University Hospital Basel, University of Basel Basel Switzerland; ^3^ Division of Genetics, Department of Cellular and Molecular Biology and Microbiology, Faculty of Science and Technology University of Isfahan Isfahan Iran; ^4^ Molecular and Clinical Sciences Institute, St. George's, University of London London UK; ^5^ Human Genetics Program Sanford Burnham Prebys Medical Discovery Institute La Jolla California USA; ^6^ Pediatrics Endocrinology Department Mashhad University of Medical Sciences Mashhad Iran; ^7^ Pediatric Neurology Department Ghaem hospital, Mashhad University of Medical Sciences Mashhad Iran; ^8^ Karyogen Medical Genetics Laboratory Alzahra University Isfahan Iran; ^9^ Child Growth and Development Research Center Research Institute for Primordial Prevention of Non‐Communicable Disease, Isfahan University of Medical Sciences Isfahan Iran; ^10^ Neuromuscular and Neurogenetic Disorders of Childhood Section National Institute of Neurological Disorders and Stroke, National Institutes of Health Bethesda Maryland USA; ^11^ Department of Anatomy, Genetics & Biomedical Informatics, Faculty of Medicine University of Colombo Colombo Sri Lanka; ^12^ Department of Pediatrics and Adolescent Medicine University Hospital Freiburg, Freiburg University Faculty of Medicine Germany; ^13^ Autism & Developmental Medicine Institute, Genomic Medicine Institute, Department of Radiology Diagnostic Medicine Institute Danville Pennsylvania USA

**Keywords:** congenital disorders of glycosylation (CDG), DPM3, dystroglycanopathy, muscle dystrophy, muscle‐eye‐brain (MEB) disease

## Abstract

Biallelic pathogenic variants in the genes encoding the dolichol‐phosphate mannose synthase subunits (*DPM*) which produce mannosyl donors for glycosylphosphatidylinositols, *N*‐glycan and protein *O*‐ and *C*‐mannosylation, are rare causes of congenital disorders of glycosylation. Pathogenic variants in *DPM1* and *DPM2* are associated with muscle–eye–brain (MEB) disease, whereas *DPM3* variants have mostly been reported in patients with isolated muscle disease—dystroglycanopathy. Thus far, only one affected individual with compound heterozygous *DPM3* variants presenting with myopathy, mild intellectual disability, seizures, and nonspecific white matter abnormalities (WMA) around the lateral ventricles has been described. Here we present five affected individuals from four unrelated families with global developmental delay/intellectual disability ranging from mild to severe, microcephaly, seizures, WMA, muscle weakness and variable cardiomyopathy. Exome sequencing of the probands revealed an ultra‐rare homozygous pathogenic missense *DPM3* variant NM_018973.4:c.221A>G, p.(Tyr74Cys) which segregated with the phenotype in all families. Haplotype analysis indicated that the variant arose independently in three families. Functional analysis did not reveal any alteration in the *N*‐glycosylation pathway caused by the variant; however, this does not exclude its pathogenicity in the function of the DPM complex and related cellular pathways. This report provides supporting evidence that, besides *DPM1* and *DPM2*, defects in *DPM3* can also lead to a muscle and brain phenotype.

## Graphical abstract

We report three unrelated Iranian families and one from Sri Lanka with five children presenting with congenital muscle weakness, developmental delay/intellectual disability, epilepsy, and white matter abnormalities due to an ultra‐rare homozygous missense *DPM3* variant. (created with biorender.com).

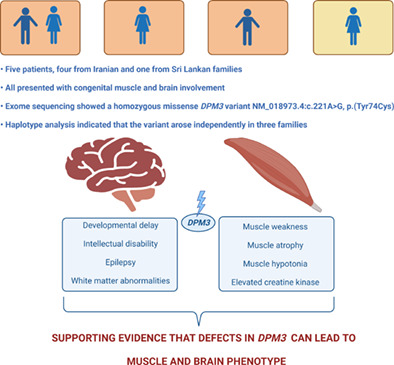

## 1 INTRODUCTION

Biallelic variants in DPM subunits 1 and 2 (*DPM1*, MIM 608799*; DPM2*, MIM 615042) are known to be associated with muscle–eye–brain (MEB) disease,^1^ while biallelic variants in *DPM3* (MIM 612937) were initially described only in the context of muscle dystrophy and cardiomyopathy.^2^, ^3^, ^4^, ^5^, ^6^ In 2019, Fu et al^7^ presented the first patient, a Chinese girl with combined muscle and central nervous system involvement due to compound heterozygous variants in *DPM3*. Here we report three unrelated Iranian families and one from Sri Lanka with five children presenting with congenital muscle weakness, developmental delay (DD)/intellectual disability (ID), and epilepsy due to an ultra‐rare homozygous missense *DPM3* variant. Informed consent was obtained from the parents.

## 2 CLINICAL CHARACTERISATION

### 2.1 Patients 1 and 2

**FIGURE 1:**
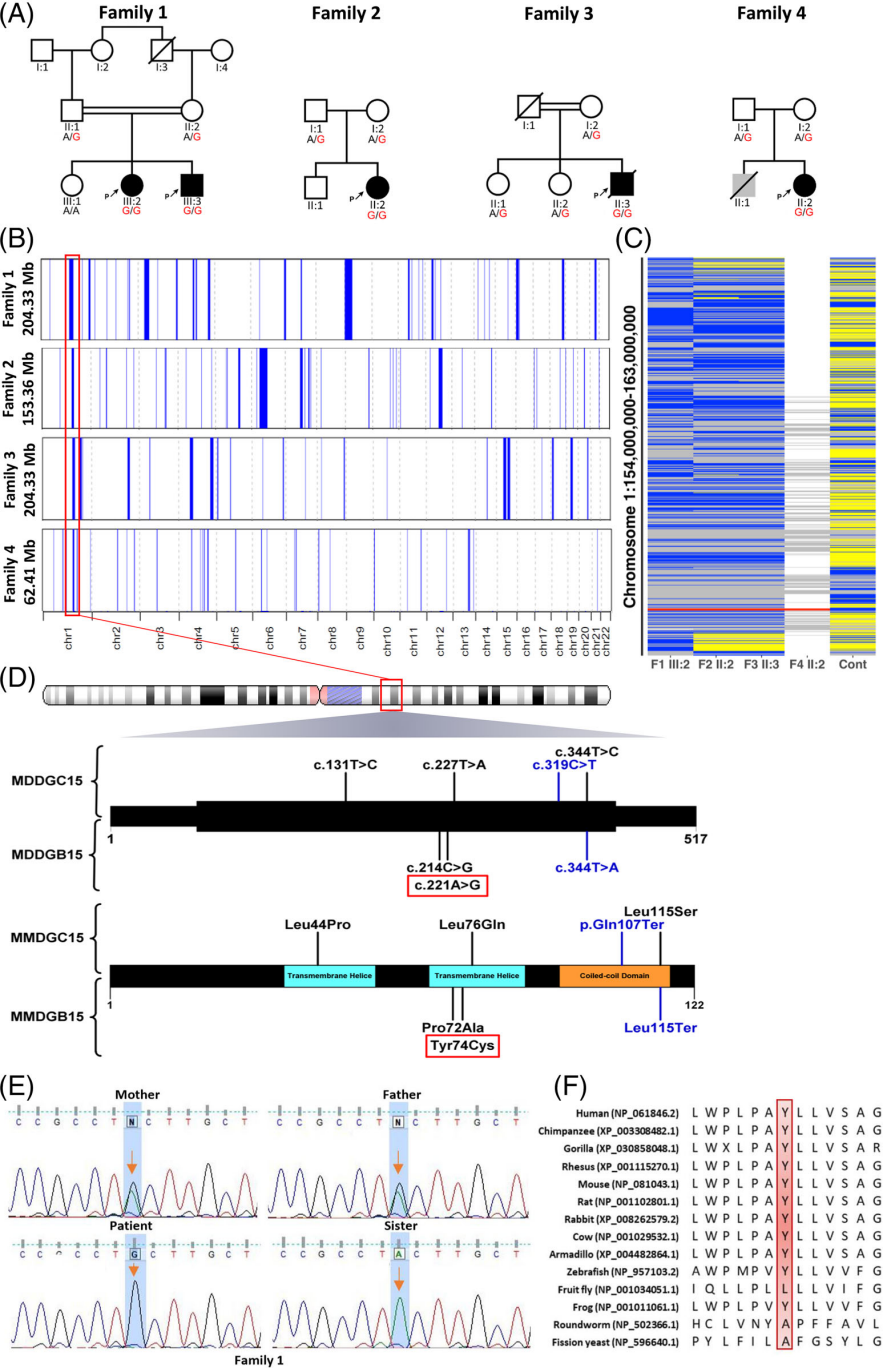
Genetic and biochemical characteristics of *DPM3* variants. (A) Family pedigrees and segregation analysis of the *DPM3* variant in 4 unrelated families. (B) Overview of the whole regions of homozygosity (ROH) in the exome of each patient and total size of ROH for each proband.^8^ The block of homozygosity surrounding the *DPM3* variant is indicated in red. (C) Pictorial representation of exome genotype data across ~9 Mb of chromosome 1q encompassing *DPM3* variant (indicated by red bar). (D) Localisation of the gene on chromosome 1q22 with all known variants and the related amino acid changes across the gene and protein. Black indicates missense, blue nonsense variants, and red box the variant identified in this study. MDDGB15 indicates muscular dystrophy‐dystroglycanopathy (congenital with impaired intellectual development), type B, 15, while MDDGC15 muscular dystrophy–dystroglycanopathy (limb‐girdle), type C, 15.^9^ (E) Sanger sequencing chromatograms from genotyping of all members of family 1. (F) Conservation of the NM_018973.4 (DPM3): c.221A > G, p.(Tyr74Cys) variant across species at protein level. [Colour figure can be viewed at wileyonlinelibrary.com]

**FIGURE 2:**
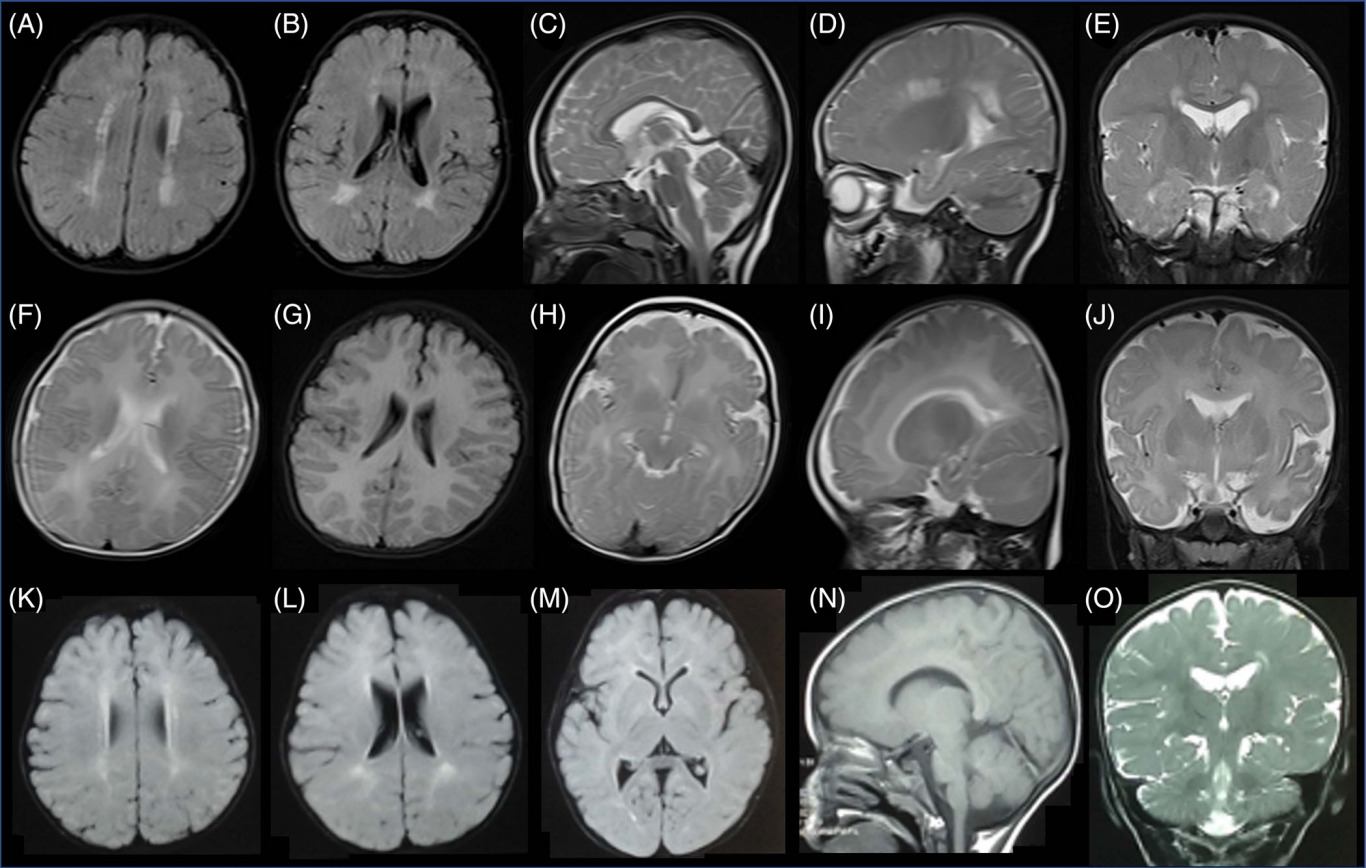
Brain MRI findings of patients with *DPM3* variants. Findings of patient 1 (A–E) include T2/FLAIR hyperintense foci in the subcortical and periventricular white matter (A, B, D, E), mildly prominent ventricles secondary to white matter volume loss (B, C, E), and corpus callosum hypoplasia (C). Findings of patient 2 (F–J) include hypomyelination, T2 hyperintense foci in the periventricular white matter (F, H, I, J), mildly prominent lateral ventricles (G, J), and slightly prominent frontal and temporal subarachnoid spaces (H, J). Findings of patient 5 (K‐O) include T2/FLAIR hyperintense foci in the subcortical and periventricular white matter (K, L) and globi pallidi (M), mildly prominent lateral ventricles, white matter volume loss (L, O), and mega cisterna magna (N). [Colour figure can be viewed at wileyonlinelibrary.com]

The proband is a 4‐year‐old girl from a consanguineous Iranian Turkmen family (Figure 1A, Family 1). She exhibited microcephaly, hypotonia, significant motor and speech delay and failure to thrive. She was able to sit unassisted at the age of 2 years, but never reached the walking milestone. She could not speak more than two words and displayed severe global DD, muscle weakness and muscle atrophy, febrile convulsions and afebrile tonic seizures. She had urinary and bowel incontinence. Blood tests revealed elevated serum transaminases (TA) and creatine kinase (CK) up to 1500 U/L. Her electroencephalogram (EEG) showed scattered sharp waves. Nerve conduction studies were normal; however, myography of the distal and proximal lower limbs showed myopathic changes without signs of acute denervation. Magnetic resonance imaging (MRI) of the brain performed at the age of 2 years showed subcortical and periventricular white matter abnormalities (WMA) most pronounced in the periatrial regions with some of the foci distributed along the axis of medullary veins perpendicular to the body of the lateral ventricles/corpus callosum (Figure 2A–E). Ophthalmologic and cardiologic examinations were normal.

In the same family, the younger brother of the proband showed a similar clinical presentation. His brain MRI performed at the age of 1 year revealed hypomyelination and periventricular WMA (Figure 2F–J).

### 2.2 Patient 3

The 2.5‐years‐old daughter of non‐consanguineous Persian parents from the same village without a family history of neurological disorders (Figure 1A, Family 2) showed microcephaly, hypotonia, muscle weakness and epilepsy as an infant, delayed motor and speech development, and dysarthria. She reached the walking milestone (with some ataxia) at the age of 2 years and 3 months. Serum CK and TA were increased, and the EEG showed a mildly abnormal background with epileptic discharges. Echocardiography revealed a mildly dilated left ventricle with mildly decreased left ventricular systolic function. There were no signs of eye involvement.

### 2.3 Patient 4

An adult Persian man born to a consanguineous marriage (Figure 1A, Family 3) presented with DD, ID, motor disability, cardiomyopathy and epilepsy. He had a progressive disease course, became wheelchair‐bound and died at the age of 26.

### 2.4 Patient 5

The proband is a 4‐year‐old Sri Lankan daughter of non‐consanguineous parents. She presented with global DD, recurrent generalised seizures since infancy, muscle weakness, hypotonia and microcephaly. She was able to sit unassisted at the age of 3 years, but never arrived at walking. Serum CK and ammonia levels were elevated with 1507 U/L and 53 μmol/L, respectively. The EEG showed changes suggestive of generalised epilepsy, and the myography revealed myopathic features. MRI scan of the brain showed T2/FLAIR hyperintense foci in the subcortical and periventricular WM and globi pallidi, mildly prominent lateral ventricles, WM volume loss and mega cisterna magna. The muscle biopsy confirmed myopathic changes with increased fibre size variability, fibre splitting and intrafascicular fibrosis (Figure 2K–O).

An affected male sibling died at the age of 1 year and 9 months from a similar but more severe condition.

## 3 GENETIC FINDINGS

Solo exome sequencing of the affected Iranian individuals and trio exome sequencing of the Sri‐Lankan family revealed the same missense variant chr1:g.155140110T>C;NM_018973.4(DPM3):c.221A>G, p.(Tyr74Cys) residing within sizable regions of homozygosity (Figure 1B). However, three distinct haplotypes surrounding the variant were found in these families (Figure 1C). Sanger sequencing segregation analysis showed the variant segregated well within the families (Figure 1E). Only seven carriers were observed in around 1.500.000 alleles aggregated across multiple variant frequency databases. The ultra‐rare variant located within one of the transmembrane domains, affecting a highly conserved residue among vertebrates (CADD: 32, GERP: 2.66, REVEL: 0.9049) (Figure 1D,F). In addition, multiple in silico tools predict the variant to being deleterious (SIFT: 0.04, PolyPhen: 0.993, Mutation Taster: 1, Provean: −5.28). The variant was classified as pathogenic according to the American College of Medical Genetics and Genomics and the Association for Molecular Pathology standards and guidelines for the interpretation of sequence variants.^6^

To assess the potential pathogenic effects of the p.(Tyr74Cys) variant on DPM3 function, patient derived fibroblasts were used to analyse lipid‐linked oligosaccharide (LLO) synthesis. Metabolic labelling of control and patient fibroblasts with ^3^H‐mannose followed by LLO extraction showed no significant difference between control and patient (Figure S1). We also attempted to analyse the potential effects of the variant on alpha‐dystroglycan (alphaDG) using the glycoconjugate specific antibody, IIH6. However, we were unable to detect alphaDG using IIH6 in patient or control fibroblast samples (Figure S2). This is likely due to the low expression level of alphaDG in fibroblasts.

## 4 DISCUSSION

Congenital muscle dystrophies caused by *DPM* variants are autosomal recessive disorders belonging to the group of dystrophy–dystroglycanopathies with limb girdle involvement. In the recent years, an increasing number of variants have been described with expansion of the phenotypes associated with *DPM1*, *DPM2* and *DPM3*. Affected individuals with *DPM* variants are, however, still rare, and thus far, only 21 patients have been reported with a pathogenic or likely pathogenic variant in one of the three subunit coding genes.^10^

Affected individuals with *DPM1* and *DPM2* variants frequently show central nervous system involvement,^1^ thus, one would expect a similar phenotype in *DPM3*. Nevertheless, only one individual has been reported with *DPM3*‐related dystroglycanopathy and WMA on brain MRI,^7^ and Lefeber et al. reported stroke‐like episodes in a patient with DPM3‐related muscle dystrophy.^2^ Hereby we describe five patients with a genetically confirmed homozygous variant in *DPM3*, presenting with muscle involvement along with WMA, DD/ID, and epilepsy. Interestingly, disease severity was variable and affected individuals exhibited distinctive features despite carrying the identical missense variant across the families (Table 1).

**TABLE 1:** clinical features of affected individuals with *DPM3*‐related disorder

		F1—Case 1	F1—Case 2	F2—Case 3	F3—Case 4^a^	F4—Case 5	Case 6 (Fu et al, 2019)
Age		4y	2y	2.5y	25y	4y	8y
Sex		f	m	f	m	f	f
Consanguinity		+	+	−	−	−	
Peripheral nervous system involvement	Muscle weakness	+	−	+	+	+	−
	Muscle atrophy	+	na	na	na	+	na
	Muscle hypotonia	+	na	+	−	+	−
Central nervous system involvement	Developmental delay	+ (Severe)	+	+ (Mild)	+	+	+
	Intellectual disability	na	na	na	+	na	+ (Mild)
	Epilepsy	+ (Febrile, tonic seizures)	+	+	+	+ (Generalised)	+ (Absence)
	Gait ataxia	−	−	+	−	−	−
	Autonomic involvement	+ (Bowel and urinary incontinence)	na	+ (Constipation)	na	−	−
Growth and development	Weight at last visit	<3rd %ile	na	Normal	na	<3rd %ile	na
	Height at last visit	Normal	na	Normal	na	<3rd %ile	na
	Microcephaly	at 15th %ile	+	<15th %ile	na	<3rd %ile	na
	Ability to walk at last visit	−	na	+	−	−	+
Brain MRI		Patchy and confluent WMA in the subcortical and periventricular regions, mild WM volume loss	Hypomyelination, WMA in the periventricular region, mildly prominent subarachnoid spaces overlying the frontal and temporal lobes	na	na	T2/FLAIR hyperintense foci in the subcortical and periventricular WM and globi pallidi, mildly prominent lateral ventricles, WM volume loss, mega cisterna magna	WMA around lateral ventricles
Elevated CK		+	na	na	na	+	+
Electromyography		Myopathic changes	na	na	na	Myopathic changes	na
Cardiac assessment		Normal	na	Cardiomyopathy	Cardiomyopathy	Normal	Normal
Ophthalmic assessment		Normal	na	normal	na	na	na
Muscle biopsy		na	na	na	na	Myopathic changes	Mild non‐specific Myopathic changes
Genetic testing		Solo whole exome sequencing and sanger sequencing				Trio whole exome sequencing	Gene panel
Variants		Homozygous c.221A > G p.(Tyr74Cy)					Compound heterozygous c.214C>G p.(Pro72Ala) c.344T>A p.(Leu115Ter)

Abbreviations: %ile, percentile; CK, creatine kinase; f, female; m, male; MRI, magnetic resonance imaging; na, not available; WM(A), white matter (abnormalities).

^a^ Deceased.

Although only one patient underwent muscle biopsy, the presence of severe muscle weakness and hypotonia suggests an underlying congenital muscle dystrophy, and in two patients, myopathic changes were confirmed by myography. Additionally, two patients presented with cardiomyopathy. The brain MRI of the patients showed periventricular WMA similar to the patient of Fu et al.,^7^ however, we also identified WMA in the subcortical region and WM volume loss. Thus, we conclude this new and pathogenic variant in *DPM3* can lead to congenital muscle and brain disease.

The exact pathogenicity of the disorder is not well understood. Glycosylation involves the addition of glycans to proteins and lipids through one of the eight major enzymatic pathways described in mammals.^10^, ^11^ The DPM complex plays a role in four of these pathways.^2^ While DPM1 is the catalytic subunit localised in the cytoplasm, DPM2 and DPM3 subunits anchor DPM1 to the endoplasmic reticulum membrane. The coiled‐coil domain of DPM3 is responsible for the anchoring of DPM1, whereas its N‐terminal transmembrane domain is linked to DPM2.^2^, ^11^, ^12^ Interestingly, the homozygous p.(Tyr74Cys) variant is located on a transmembrane domain next to p.(Pro72Ala) variant which was identified as compound het with p.(Leu115Ter) in the girl with muscle and brain presentation.^7^ However, the biochemical assay could not detect any changes in the *N*‐glycosylation pathway, which correlates with previous findings stating that the transmembrane domain is not necessary for the enzymatic activity of the complex.^12^ In contrast, the highly conserved coiled‐coil region was shown to be required for the enzymatic process, and variant in this domain was associated with reduced *O*‐mannosylation of alpha‐dystroglycan due to reduced binding capacity of DPM3 for DPM1.^2^ The finding that the variant we identified does not seem to disturb glycosylation points to potentially other mechanisms of pathogenicity. The haplotype analysis suggests that the *DPM3* variant most likely recurred in at least three families which was not unexpected given the different ethnicities of the families. This finding along with consistent clinical and genetic data across four independent families provides additional support that p.Tyr74 residue is fundamental for proper function of DPM3.

In this study, we consolidate the association of *DPM3* variants with brain‐muscle phenotype and further delineate the molecular and clinical spectrum associated with this new ultra‐rare congenital disorder of glycosylation.

## AUTHOR CONTRIBUTIONS

Sara Nagy reviewed the clinical data and drafted the manuscript. Tracy Lau, Vajira H.W. Dissanayake, and Nirmala D. Sirisena reviewed the genetic data. Ehsan Ghayoor Karimiani, Jalal Vallian, Shahryar Alavi, Samaneh Noroozi Asl, Javad Akhondian, Amir Bahreini, Omid Yaghini, Vajira H.W. Dissanayake, and Nirmala D. Sirisena provided clinical information. Miriam Schmidts, Jalal Vallian, Shahryar Alavi, Prech Uapinyoying and Carsten Bonnemann performed genetic testing. Bobby G. Ng and Hudson H. Freeze performed the functional assay. Andres Moreno‐De‐Luca reviewed the imaging data. Henry Houlden edited the manuscript. Reza Maroofian supervised the project and edited the manuscript.

## CONFLICT OF INTEREST

The authors have no conflict of interest.

## ETHICS STATEMENT

All participated families provided informed consent. Procedures were reviewed and approved by the appropriate institutional review committee.

## Supporting information


**Figure S1** High‐performance liquid chromatography (HPLC) analysis of lipid linked oligosaccharide (LLO) in control (black solid line) and patient fibroblasts (red dash line) labelled with 3H‐mannose. LLO samples were separated by HPLC and fractions were collected and counted on a scintillation counter machine.

**Figure S2** Fibroblast cell extracts from control and patient samples were used to purify WGA reactive proteins and then run‐on a SDS page gel. The glycosylated form of α‐DG was detected using the monoclonal α‐DG clone IIH6. Samples were run in biological duplicates.
GM 00038, GM 05565, GM 09503: healthy controls; CDG 0127: patient sample.

## Data Availability

Anonymized data from participants will be available on request.
