# Supplementary figures and images for: A recurrent homozygous missense DPM3 variant leads to muscle and brain disease
Source: Clin Genet. 2022 Aug 19;102(6):530–6. doi: 10.1111/cge.14208 (PMC9633384; doi:10.1111/cge.14208)

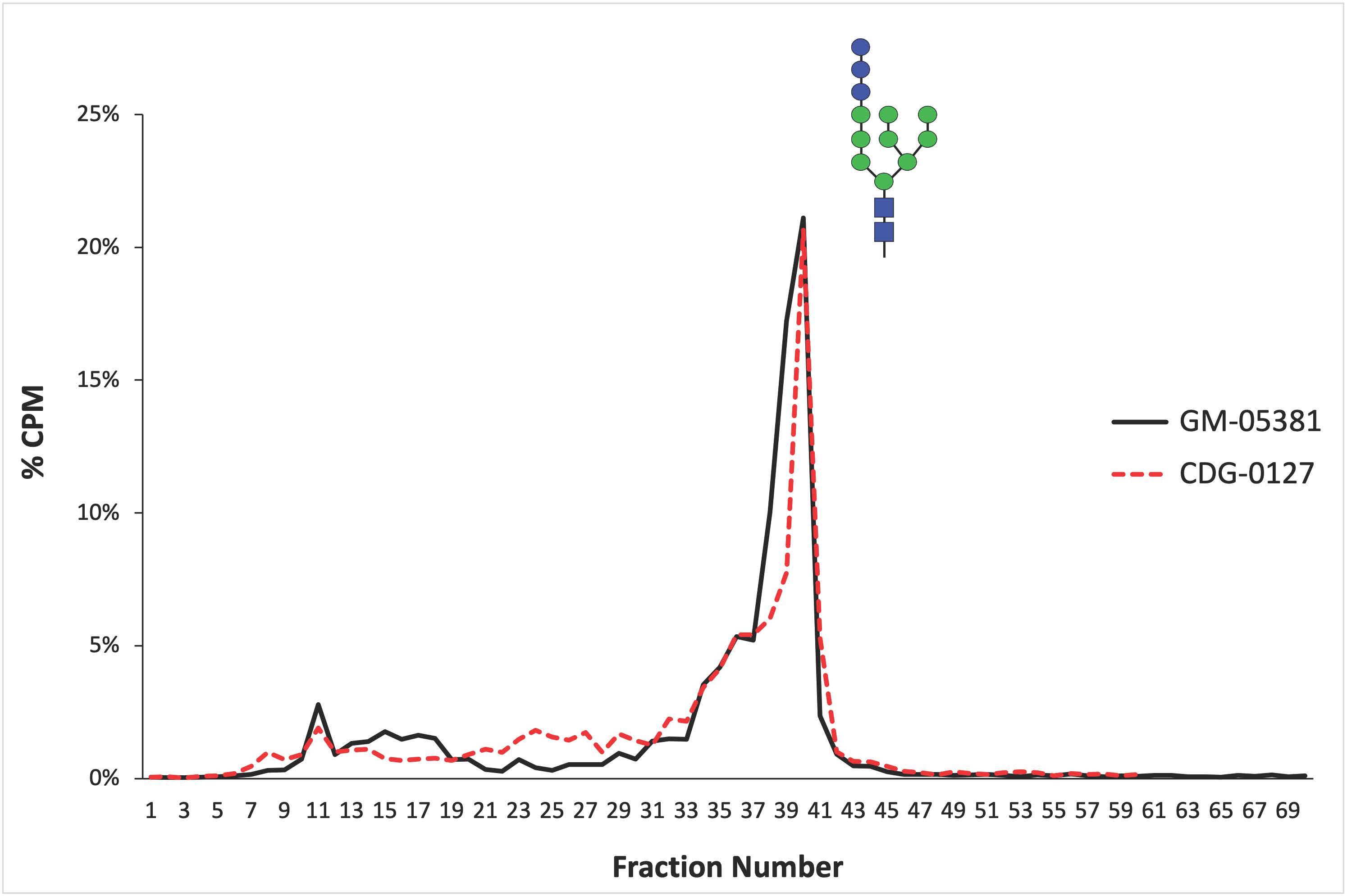

Supplement: Supplementary file 1 — Figure S1 High‐performance liquid chromatography (HPLC) analysis of lipid linked oligosaccharide (LLO) in control (black solid line) and patient fibroblasts (red dash line) labelled with 3H‐mannose. LLO samples were separated by HPLC and fractions were collected and counted on a scintillation counter machine. [file CGE-102-530-s001.jpg]

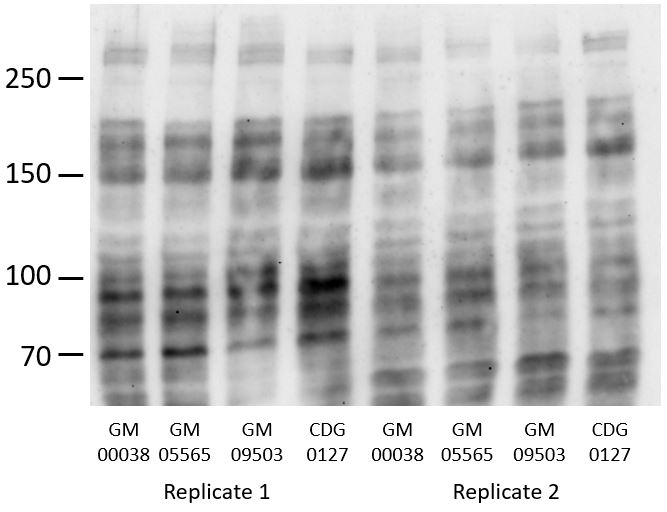

Supplement: Supplementary file 2 — Figure S2 Fibroblast cell extracts from control and patient samples were used to purify WGA reactive proteins and then run‐on a SDS page gel. The glycosylated form of α‐DG was detected using the monoclonal α‐DG clone IIH6. Samples were run in biological duplicates. GM 00038, GM 05565, GM 09503: healthy controls; CDG 0127: patient sample. [file CGE-102-530-s002.JPG]
